# Concomitant use of tamoxifen with radiotherapy enhances subcutaneous breast fibrosis in hypersensitive patients

**DOI:** 10.1038/sj.bjc.6602146

**Published:** 2004-08-24

**Authors:** D Azria, S Gourgou, W J Sozzi, A Zouhair, R O Mirimanoff, A Kramar, C Lemanski, J B Dubois, G Romieu, A Pelegrin, M Ozsahin

**Affiliations:** 1 Department of Radiation Oncology, Centre Régional de Lutte contre le Cancer, Rue Croix Verte, 34298 Montpellier, France; 2 EMI 0227, Centre Régional de Lutte contre le Cancer, Rue Croix Verte, 34298 Montpellier, France; 3 Biostatistics Unit, Centre Régional de Lutte contre le Cancer, Rue Croix Verte, 34298 Montpellier, France; 4 Department of Radiation Oncology, Centre Hospitalier Universitaire Vaudois, Rue Bugnon, Lausanne, Switzerland; 5 Department of Medical Oncology, Centre Régional de Lutte contre le Cancer, Rue Croix Verte, 34298 Montpellier, France

**Keywords:** subcutaneous fibrosis, breast cancer, tamoxifen, radiotherapy, hypersensitive patients

## Abstract

Concomitant use of adjuvant tamoxifen (TAM) and radiation therapy (RT) is not widely accepted. We aim to assess whether this treatment is associated with an increased risk of developing subcutaneous fibrosis after conservative or radical surgery in breast cancer patients. We analysed 147 women with breast cancer treated with adjuvant RT, and who were included in the KFS 00539-9-1997/SKL 00778-2-1999 prospective study aimed at evaluating the predictive value of CD4 and CD8 T-lymphocyte apoptosis for the development of radiation-induced late effects. TAM (20 mg day^−1^) with concomitant RT was prescribed in 90 hormone receptor-positive patients. There was a statistically significant difference in terms of complication-relapse-free survival (CRFS) rates at 3 years, 48% (95% CI 37.2–57.6%) *vs* 66% (95% CI 49.9–78.6%) and complication-free survival (CFS) rates at 2 years, 51% (95% CI 40–61%) *vs* 80% (95% CI 67–89%) in the TAM and no-TAM groups, respectively. In each of these groups, the CRFS rates were significantly lower for patients with low levels of CD8 radiation-induced apoptosis, 20% (95% CI 10–31.9%), 66% (95% CI 51.1–77.6%), and 79% (95% CI 55–90.9%) for CD8 ⩽16, 16–24, and >24%, respectively. Similar results were observed for the CFS rates. The concomitant use of TAM with RT is significantly associated with an increased incidence of grade 2 or greater subcutaneous fibrosis; therefore, caution is needed for radiosensitive patients.

The efficacy of radiotherapy (RT) in the treatment of malignant disease is limited by the need to avoid severe and nonreversible late damage to normal tissues. Nevertheless, the positive impact of RT in several tumours such as breast cancer makes its use inevitable. Indeed, postoperative RT decreases the risk of locoregional recurrence and is associated with improved survival in high-risk premenopausal and postmenopausal breast cancer patients given adjuvant chemotherapy or tamoxifen (TAM), respectively (Overgaard *et al*, 1997, 1999).

The use of adjuvant TAM in postmenopausal breast carcinoma patients as an adjunct to primary surgery is well established. The benefits from this treatment have been shown in lymph node-negative as well as lymph node-positive patients, both in terms of a prolonged recurrence-free survival and an increase in overall survival, especially in women presenting with oestrogen receptor-positive tumours (EBCTCG *et al*, 1998). The role of TAM is still under discussion in ductal carcinoma *in situ* of the breast after complete resection (Fisher *et al*, 1999; Houghton *et al*, 2003).

The interaction of TAM and RT remains poorly defined. TAM appears to exert its cytostatic activity at least partly through competitive inhibition at the oestrogen receptor, resulting in segregation of cells into G_0_/G_1_ phase of the cell cycle (Osborne *et al*, 1983). Because relatively less radiosensitivity has been observed in early G_1_ (Sinclair, 1968), a hypothetical concern is raised with TAM whether its combination with radiotherapy results in the radioprotection of tumour clonogens of hormonally responsive and unresponsive breast carcinoma cells at dose levels typical of those used clinically (Wazer *et al*, 1989). However, in this study, the cell cultures were grown in a medium containing phenol red and foetal bovine serum, two sources of exogenous oestrogenic compounds (Butler *et al*, 1981; Page *et al*, 1983; Berthois *et al*, 1986; Germain and Harbrioux, 1993). This fact complicates the interpretation of the resultant radiation survival curves (Gould and Clifton, 1978). In contrast to these reports, no significant differences were observed in terms of radiosensitivity for oestradiol-stimulated or 4-hydroxytamoxifen-inhibited cultures plated under growth-stimulating conditions immediately after irradiation or following an additional 24 h under oestrogen-free conditions (Sarkaria *et al*, 1994). Clearly, under defined hormonal conditions (Cormier and Jordan, 1989), no protective effect of the active TAM metabolite, 4-hydroxytamoxifen, was observed (Sarkaria *et al*, 1994). In addition, irradiation and TAM may modify the oestrogen and progesterone receptor content in the cytosol in breast cancer cells and that could explain their change in radiation sensitivity (Paulsen *et al*, 1996). Results from a study in tumour-bearing rats (Kantorowitz *et al*, 1993) receiving fractionated RT, TAM, or both showed that, in contrast to *in vitro* results, the combination treatment reduced the probability of subsequent tumour development.

In so far as the preclinical data with breast cancer cells can be extrapolated to the clinical situation, no alteration in responsiveness would be expected following TAM exposure. Although no clinical trials have been designed to address specifically the effect of concurrent TAM on the response to conventional RT, results from clinical trials which included treatment arms with and without TAM suggest that no deleterious consequences accompanied TAM treatment. Results from the National Surgical Adjuvant Breast Project (NSABP)-B14 trial suggested that TAM and RT may have a synergistic interaction since patients receiving both therapies experienced a higher probability of local control (Fisher *et al*, 1989). In the Danish Breast Cancer Cooperative Group 82c randomised trial, RT and TAM were associated with a lower risk of locoregional recurrence and improved survival in high-risk postmenopausal breast cancer patients after mastectomy and limited axillary dissection, after only 1 year of adjuvant TAM treatment (Overgaard *et al*, 1999). However, TAM has been reported to lead to worse cosmesis in women who underwent conservative surgery, RT, and had received TAM (Wazer *et al*, 1992) but not in others (Taylor *et al*, 1995; Fowble *et al*, 1996). Wazer *et al* (1997) found no adverse effect of TAM on cosmesis in an updated analysis of 498 women who were treated with breast-conserving therapy. Adjuvant tamoxifen was administered to 130 patients, beginning 1–6 weeks after irradiation. More extensive regional lymphatic irradiation was administered to the TAM+ group. Chemotherapy was administered to 15% of TAM+ and 28% (*P*=0.003) of TAM− patients. There were no significant differences between the groups with respect to tumour size, re-iexcision, total excised tissue volume, final margin status, total radiation dose, or use of interstitial implant boost. There was no significant difference between the TAM+ and TAM− groups in the overall distribution of cosmetic scores (*P*=0.18). The 5-, 7-, and 10-year actuarial local failure rates for TAM+ *vs* TAM− patients were 0 *vs* 3.1%, 1.9 *vs* 5.4%, and 1.9 *vs* 8.4%, respectively. Multivariate regression analyses of potentially confounding variables revealed no significant associations between tamoxifen and either cosmetic outcome or local failure.

Our goal in this study was to evaluate the relationship between the concomitant administration of TAM and adjuvant RT and the risk of developing subcutaneous fibrosis after conservative or radical surgery in breast cancer patients. The analysis was based on patients participating in a large prospective study of 399 patients where we evaluated the correlation between the level of radiation-induced apoptosis of CD4 and CD8 lymphocytes and late side effects (Ozsahin *et al*, 2003).

## PATIENTS AND METHODS

### Design of the study

All patients included in the KFS 00539-9-1997/SKL 00778-2-1999 prospective study (Ozsahin *et al*, 2003) were used in evaluating the predictive value of CD8 T-lymphocyte apoptosis on the development of radiation-induced late side effects, notably fibrosis. Among the 399 patients, 147 women presented with breast cancer. Our objective was to assess, in this population, whether the risk of developing subcutaneous fibrosis after conservative or radical surgery and adjuvant RT was increased by the concomitant administration of TAM.

### Radiation-induced apoptosis

Heparinised whole blood (7 ml) was obtained from consenting patients participating in the study, diluted 1 : 10 in RPMI 1640 medium (Life Technologies, Basel, Switzerland) containing 20% foetal bovine serum (Readysysteme, Zurzach, Switzerland), and was divided into two 3.5-ml aliquots and placed in 25-cm^2^ (60 ml) flasks. These aliquots were irradiated at room temperature under oxic conditions with 0- and 8 Gy using an Oris IBL 137 cesium source (CIS-Bio International, Gif-sur-Yvette, France) at a dose rate of 2.67 cGy s^−1^. Following irradiation, the preparations were incubated at 37°C in 5% CO_2_. After 48 h, the contents of each flask were distributed into four 5-ml test tubes and then centrifuged at 1300 r.p.m. for 5 min at room temperature. Most of the supernatant was aspirated and the pellet resuspended in approximately 200 *μ*l of the remaining solution. A volume of 10 *μ*l of FITC-conjugated anti-CD8 monoclonal antibody were added (Becton-Dickinson, Basel, Switzerland). Following incubation for 20 min at room temperature, 4 ml of 1 : 10 diluted lysis solution (Becton Dickinson, Basel, Switzerland) was added to the suspension, and the specimens were left for 10 min at room temperature in the dark to promote lysis of erythrocytes. The cells were then centrifuged at 1450 r.p.m. for 5 min, the supernatant was aspirated, and the cells were washed once with 4 ml phosphate-buffered saline (PBS; Becton Dickinson, Basel, Switzerland). After another round of centrifugation (1450 r.p.m. for 5 min), the supernatant was aspirated. The cells were resuspended in 200 *μ*l of FACSFlow (Becton Dickinson, Basel, Switzerland) phosphate buffer to which 5 *μ*l of propidium iodide (PI) stock (1 mg ml^−1^ in PBS) was added to stain the DNA. Then, 50 *μ*l of RNase stock solution (1 mg ml^−1^) was added, and the samples were incubated at room temperature for 5 min before flow cytometric measurement. Samples were measured using a FACScan flow cytometer (Becton-Dickinson, San Jose, CA, USA) with a 488 nm, 15 mW argon-ion laser (Coherent, Santa Clara, CA, USA). Data analysis were performed via a two-step procedure using the CellQuest software (Becton-Dickinson, Basel, Switzerland) on a Macintosh computer. Data from each lymphocyte sample were acquired immediately after the staining procedure. Four-parameter acquisition permitted discrimination of the different subpopulations of lymphocytes. Forward and side light scattering and stain-induced fluorescence at two different wavelengths (530 nm green, and 640 nm red) were simultaneously measured from each cell. Using forward scatter (FSC) *vs* side scatter (SSC) dot plots, three subpopulations of leukocytes (mono-, granulo-, and lymphocytes) as well as the cell debris could be distinguished, and the lymphocytes were selected. After staining the cells with FITC-conjugated antibodies (green fluorescence) to identify lymphocyte cell-type and PI (red fluorescence) to quantify cellular DNA content, the CD8-positive cells were identified by simultaneous measurement of the two laser-induced fluorescent signals. Apoptotic lymphocytes were defined as those cells staining positively for their cell-type-specific antibodies, and displaying reduced DNA content and cell size. These lymphocytes were previously examined for apoptotic cells by the TUNEL assay (Ozsahin *et al*, 1997). Data for at least 10 000 cells sample^−1^ were acquired.

### Treatment modalities

All patients had staging investigations including chest X-ray, bone scan, and liver enzymes to rule out metastatic disease at diagnosis. Initial values of age, TNM 2002 staging according to the American Joint Committee on Cancer staging system for breast cancer (Singletary *et al*, 2002), histopathology, type of surgery, margins, and menopausal status were noted. Surgical treatment consisted of breast-conserving surgery (any type) or mastectomy and axillary dissection in 118 (80.3%) and 29 patients, respectively.

In those patients having breast-conserving surgery, irradiation was delivered to the breast and, when indicated, to the regional lymphatics. Treatment portals consisted of opposing tangential fields using cobalt or 6–18 MV photons. Both tangential fields were treated daily. A physical lead block or asymmetrical collimation was used for half-beam blocking. The breast dose, routinely prescribed to the midline, was 50 Gy at 2 Gy fractions, with a varying percentage of compensating filters and/or bolus. All patients received a 16-Gy boost (20 Gy in the case of suspicious surgical margins) to the primary tumour bed using 6–15 MeV electrons.

The 29 mastectomy patients received chest-wall irradiation using opposing tangential fields with cobalt or 6–18 MV photons using half-beam blocking. Both tangential fields were treated daily. The chest wall dose was 50 Gy at 2 Gy fractions using compensating filters and/or bolus (one-third to half of the treatment). A 4–6 MeV electron boost of 10–16 Gy was given to the surgical scar in high-risk patients. Nodal irradiation, when indicated (mostly supraclavicular and internal mammary nodes), was given at a total dose of 50 Gy in 25 fractions. Supraclavicular lymph nodes were treated with a split anterior oblique (10–15°) beam at a dose of 50 Gy calculated at the depth of 3 cm. Internal mammary nodal irradiation in some patients was administered using a separate anterior field, namely, 25 Gy at a 4-cm depth with 6 MV photons, followed by 25 Gy at the 100% isodose line using tailored electrons (mostly 12 MeV) according to the position of the internal mammary lymph nodes as assessed on the CT-scan. When indicated, axillary lymph nodes were included in the anterior supraclavicular field, and the missing axillary midline dose was completed using a posterior axillary field.

Post- or perimenopausal women who were shown to have oestrogen receptor and/or progesterone receptor-positive tumours (ER and/or PgR=10 fmol mg^−1^ cytosol protein; or=10% of the tumour cells positive by an immunocytochemical assay) were prescribed 20 mg of tamoxifen (TAM) daily. In all cases, TAM therapy was initiated the month before or the day of the start of RT. Premenopausal women (*n*=11) with receptor-positive tumours were given 20 mg of tamoxifen daily for 5 years and an LHRH analogue monthly for at least 2 years. None of the patients who received TAM received adjuvant chemotherapy.

Women who were shown to have oestrogen receptor- and progesterone receptor- negative tumours with axillary lymph node metastasis received six cycles of chemotherapy with cyclophosphamide, fluorouracil, and methotrexate, or with cyclophosphamide and adriamycin. No treatment with concomitant chemotherapy and irradiation was given. Typically, the patients began RT 3 weeks after the completion of chemotherapy.

### Radiation-induced assessment of side effects

During treatment, acute toxicity was evaluated according to WHO and CTC-NCI v2.0 criteria. All patients were visited every 6 months for 2 years. During the follow-up visits, late side effects were graded according to the RTOG/EORTC scale (Cox *et al*, 1995). The time at which the maximal grade of late side effects was observed, that is, before 2 years had elapsed, was retained for analysis (RTOG-1). Patients were revaluated for late side effects at 2 years (RTOG-2) by a second physician (DA). The assessment of toxicity was blinded to treatment.

### Statistical analysis

Data were summarised by frequencies and percentages for categorical variables and by means, standard deviations, median, and range for continuous variables. Three categories of absolute change in the percent CD8 cells in apoptosis before and after exposure to 8 Gy of irradiation were constructed around the median value. The Kruskal–Wallis test was used to compare the continuous variables, and the *χ*^2^-test was used to compare the categorical variables between the two groups of patients with or without TAM.

All survival estimations were computed from the date of start of radiotherapy. Overall survival (OS), relapse-free survival (RFS), complication-free survival (CFS), and complication-relapse-free survival (CRFS) curves were estimated by the Kaplan–Meier method using the following first event definitions, death for OS, local or distant recurrence or death for RFS, grade 2 or 3 fibrosis for CFS, and any event for CRFS. The median follow-up was also estimated by the Kaplan–Meier method.

For OS, patients alive at the last follow-up visit were censored. For RFS, patients alive and relapse-free were censored at the last follow-up visit. For CFS, patients alive who never experienced a grade 2 or more fibrosis were censored at the last follow-up visit. Patients who relapsed before a grade 2 or greater fibrosis were censored at the time of relapse. For CRFS, patients alive and relapse-free who never experienced a grade 2 or greater fibrosis were censored at the last follow-up visit. The log-rank test was used to identify significant categorical variables for each of the survival curves. A step-wise Cox proportional hazards regression model was used for multivariate analysis. A *P*-value less than 0.05 was considered statistically significant. The data were expressed as means ±95% confidence intervals (CI). All statistical tests were two-sided.

Competing risk methodology was used to estimate the cumulative incidence of each first failure type, grade 2 or greater fibrosis, and relapse. These estimates may be different from those obtained from the inverse Kaplan–Meier survival function estimates since the event-time distributions of each failure type was taken into account rather than censored independently of the other event (Arriagada *et al*, 1992).

## RESULTS

### Patient characteristics

Patient characteristics of the 147 patients are presented in Table 1

**Table 1:** Patient characteristics

	**No tamoxifen *N*=57 (%)**	**Tamoxifen *N*=90 (%)**	**All patients *N*=147 (%)**	***P*-value**
*Age, years*				
Mean (s.d.)	55.5 (1.61)	58.7 (1.21)	57.4 (11.78)	
Median (range)	55.0 (26–80)	59.0 (35–82)	57.0 (26–82)	
<60	40 (70.2)	46 (51.1)	86 (58.5)	
⩾60	17 (29.8)	44 (48.9)	61 (41.5)	0.022
*Histopathology*^a^				
IDC	36 (63.2)	67 (74.5)	103 (70.1)	
ILC	7 (12.3)	18 (20.0)	25 (17.0)	
DCIS	10 (17.5)	3 (3.3)	13 (8.8)	
Others	4 (7.0)	2 (2.2)	6 (4.1)	0.008
*TNM*^b^				
T *in situ*	10 (17.9)	2 (2.3)	12 (8.4)	
T1	28 (50.0)	49 (56.3)	77 (53.8)	
T2	10 (17.9)	27 (31.0)	37 (25.9)	
T3	4 (7.1)	4 (4.6)	8 (5.6)	
T4	4 (7.1)	5 (5.8)	9 (6.3)	0.005^c^
N N0	40 (71.4)	46 (52.9)	86 (60.1)	
N1	12 (21.4)	39 (44.8)	51 (35.7)	
N2	2 (3.6)	—	2 (1.4)	
N3	2 (3.6)	2 (2.3)	4 (2.8)	0.027^d^
M M0	56 (100.0)	87 (100.0)	143 (100.0)	
M1	—	—	—	—
Type of surgery				
Mastectomy	12 (21.1)	17 (18.9)	29 (19.7)	
Conservative	45 (78.9)	73 (81.1)	118 (80.3)	0.748
Tumorectomy	31 (54.4)	49 (54.4)	80 (54.4)	
Quadrantectomy	14 (24.5)	24 (26.7)	38 (25.9)	0.931
*Margins*				
Clear	52 (91.2)	86 (95.6)	138 (93.9)	
Positive or close	5 (8.8)	4 (4.4)	9 (6.1)	0.286
Menopausal status				
Pre	81 (4.0)	11 (12.2)	19 (12.9)	
Peri or post	49 (86.0)	79 (87.8)	128 (87.1)	0.75

a IDC=invasive ductal carcinoma; ILC=invasive lobular carcinoma; DCIS=ductal carcinoma *in situ*.

b Initial TNM was not available for four patients but these patients completed their treatments and continued to be visited at each medical evaluation. None of them were M1 during the follow-up visits.

c Test on Tis/T1/T2/T3+T4.

d Test on N0 *vs* N1+N2+N3.

. There were significantly more patients 60 years or older in the group that received TAM than in the group that did not receive TAM (49 *vs* 30%). Patients who received TAM were significantly more likely to have had pathologically positive axillary lymph nodes (47.1 *vs* 28.6%), larger tumour size (41 *vs* 32% ⩾T2), and more invasive lobular carcinoma histopathologic subtypes (20 *vs* 12%). No difference was identified regarding margin measurements. Most of the patients were postmenopausal (87%) with no difference between the two groups. The extent of surgery applied to both groups was similar with 80% of patients having had breast-conserving surgery.

### Treatment delivery

The radiation therapy characteristics are presented in Table 2

**Table 2:** Treatment delivery and characteristics of CD8 radiation-induced apoptosis

	**No tamoxifen *N*=57**	**Tamoxifen *N*=90**	**All patients *N*=147**	***P*-value**
*Duration, days*				
Mean (s.d.)	45.4 (0.99)	46.9 (0.70)	46.3 (7.02)	
Median (range)	46.0 (17–57)	48.0 (23–70)	47.0 (17–70)	0.187
*Dose (Gy)*				
<35	1 (1.7%)	1 (1.1%)	2 (1.4%)	
50	8 (14.0%)	4 (4.4%)	12 (8.2%)	
60	1 (1.7%)	1 (1.1%)	2 (1.4%)	
66	42 (73.7%)	80 (88.9%)	122 (82.9%)	
⩾68	5 (8.8%)	4 (4.4%)	9 (6.1%)	
*Dose/fraction (Gy)*				
1.8	—	1 (1.1%)	1 (0.7%)	
2	56 (98.2%)	89 (98.9%)	145 (98.6%)	
3	1 (1.8%)	—	1 (0.7%)	
*Energy*				
Breast-conserving surgery				
Cobalt^60^	33 (73.3%)	47 (64.4%)	80 (67.8%)	
X6 (MV)	12 (26.7%)	26 (35.6%)	38 (32.2%)	0.312
Mastectomy				
Cobalt^60^	6 (50.0%)	10 (58.8%)	16 (55.2%)	
X6 (MV)	6 (50.0%)	7 (41.2%)	13 (44.8%)	0.638
Volume of the irradiated breast (ml)				
*Breast-conserving surgery*	*N*=45	*N*=73	*N*=118	
Mean (s.d.)	1134.4 (323.84)	1323.7 (613.56)	1251.5 (528.81)	
Median (range)	1071.0 (602.3–2018.3)	1224 (480–4032)	1127.0 (480–4032)	0.059
*Mastectomy*	*N*=12	*N*=17	*N*=29	
Mean (s.d.)	911.2 (394.52)	1075.9 (455.6)	1007.8 (431.9)	
Median (range)	831.4 (514.3–1930.5)	966.9 (675–2268)	900.0 (514.3–2268)	0.320
Dose of the surface of the breast (Gy)				
*Breast-conserving surgery*	*N*=45	*N*=73	*N*=118	
Mean (s.d.)	92.3 (10)	95.1 (6.6)	94.1 (8.1)	
Median (range)	94.1 (50.6–104.7)	95.4 (68.6–105.8)	95.3 (50.6–105.8)	0.067
*Mastectomy*	*N*=12	*N*=17	*N*=29	
Mean (s.d.)	95.4 (7.76)	94.6 (7.4)	94.9 (7.44)	
Median (range)	95.1 (83.2–107.2)	95.1 (79.1–107.2)	95.1 (79.1–107.2)	0.762
CD8 (before RT^*^, %)				
Mean (s.d.)	9.1 (1)	7.9 (0.73)	8.4 (7.18)	
Median (range)	6.5 (1.3–38.2)	5.6 (0.8–35.3)	6.3 (0.8–38.2)	0.134
CD8 (after 8 Gy, %)				
Mean (s.d.)	31.9 (1.74)	26.6 (1.1)	28.7 (11.8)	
Median (range)	29.7 (10.2–69.8)	26.2 (5.8–59.6)	28.2 (5.8–69.8)	0.024
CD8 Difference, %				
Mean (s.d.)	22.9 (1.31)	18.7 (1.03)	20.3 (9.96)	
Median (range)	21.8 (6.2–51.9)	17.6 (3.4–55.7)	20.0 (3.4–55.7)	0.008
CD8 (%)⩽16	13 (22.8%)	37 (41.1%)	50 (34%)	
16–24	21 (36.8%)	31 (34.4%)	52 (35.4%)	
>24	23 (40.4%)	22 (24.4%)	45 (30.6%)	0.041

. All but two patients (99%) received a dose rate of 2 Gy per fraction. The intensity of RT administered was similar for the two groups with no significant difference in the total dose of radiation, type of energy (cobalt or X-rays) delivered, volume of the irradiated breast, or the calculated dose at the surface of the breast. Median treatment duration was 47 days (range 17–70).

All patients receiving TAM were hormone receptor positive, and none received adjuvant chemotherapy. Chemotherapy was administered for hormone receptor-negative patients with positive axillary nodes and who were younger than 65 years old (12 patients, 21%). The CD8-radiation-induced apoptosis characteristics are presented in Table 2. The overall mean difference (±s.d.) before and after radiotherapy was 20.3 (±9.96) with a statistically significant difference observed between the two groups, 18.7 and 22.9 in the TAM and no TAM groups, respectively. Significantly more patients included in the TAM group had CD8 radio-induced apoptosis ⩽16% (41%) than patients not receiving TAM (23%).

### Acute toxicity

All patients experienced at least a grade 1 acute WHO side effect with 23.1% grade 3 of breast skin toxicity. No difference between the TAM+ and TAM− groups was observed. According to the CTC-NCI v2.0 classification, only five patients (3.4%) experienced grade 3 radiation dermatitis with no statistical difference between the two groups. No grade 4 toxicity was observed. Finally, neither WHO nor NCI-CTC v2.0 acute toxicities were correlated with CD8 radiation-induced apoptosis.

### Relapse-free and overall survival

The median follow-up was 29 months (range: 23–79). Ten patients relapsed (6.8%), five of whom died (3.4%). The 3-year survival rate and the relapse-free survival rates were 97% (95% CI 88**–**99%) and 91% (95% CI 81**–**95%), respectively.

### Late side effects

One patient was not evaluated for late side effects before 2 years because of early relapse. Four patients were followed up for less than 2 years and were not clinically examined for late side effects. Late side effects according to the RTOG scale are presented in Table 3

**Table 3:** RTOG late side effects

	**No tamoxifen**	**Tamoxifen**	**All patients**
*RTOG-1*	*N*=56 (%)	*N*=90 (%)	*N*=146^a^ (%)
*Skin*			
Gr0	14 (25.0)	23 (25.6)	37 (25.3)
Gr1	30 (53.6)	35 (38.9)	65 (44.5)
Gr2	12 (21.4)	31 (34.4)	43 (29.5)
Gr3	—	1 (1.1)	1 (0.7)
Gr4	—	—	—
Gr⩾2	12 (21.4)	32 (35.5)	44 (30.2)
*Subcutaneous tissue*			
Gr0	33 (58.9)	33 (36.7)	66 (45.2)
Gr1	15 (26.8)	26 (28.9)	41 (28.1)
Gr2	8 (14.3)	28 (31.1)	36 (24.6)
Gr3	—	3 (3.3)	3 (2.1)
Gr4	—	—	—
Gr⩾2	8 (14.3)	31 (34.4)	39 (26.7)
			
*RTOG-2*	*N*=56 (%)	*N*=87 (%)	*N*=143^b^ (%)
*Skin*			
Gr0	15 (26.8)	21 (24.1)	36 (25.2)
Gr1	26 (46.4)	31 (35.6)	57 (39.8)
Gr2	13 (23.2)	32 (36.8)	45 (31.5)
Gr3	2 (3.6)	3 (3.5)	5 (3.5)
Gr4	—	—	—
Gr⩾2	15 (26.8)	35 (40.3)	50 (35.0)
*Subcutaneous tissue*			
Gr0	25 (44.6)	21 (24.1)	46 (32.1)
Gr1	21 (37.5)	24 (27.6)	45 (31.5)
Gr2	10 (17.9)	31 (35.6)	41 (28.7)
Gr3	—	11 (12.7)	11 (7.7)
Gr4	—	—	—
Gr⩾2	10 (17.9)	42 (48.3)	52 (36.4)

Gr=grade; RTOG-1=the time at which the highest grade of late side effects was observed RTOG-2, late side effects at 2 years.

a One patient was not evaluated because of early relapse.

b Four patients relapsed before 2 years and were not evaluated for RTOG-2.

. A total of 135 patients (92.5%) had at least a grade 1 RTOG side effect before the first 2 years of follow-up. Four patients treated with TAM experienced early grade 3 toxicities: three subcutaneous fibrosis and one telangectasia. Among these four patients, all remained grade 3 at 2 years, and none relapsed. In all, 36 patients had grade 2 subcutaneous toxicity before 2 years. Among these patients and at 2 years, 29 remained grade 2, two decreased to grade 1, five increased to grade 3. At 2 years, 129 patients (90.2%) had at least grade 1 toxicity. Overall, 14 patients experienced 16 grade 3 skin and/or subcutaneous side effects within two years, 11 subcutaneous fibrosis and five skin side effects. Among them, two patients had both grade 3 skin and subcutaneous side effects.

### Complication-relapse-free survival

Complication-relapse-free survival according to prognostic factors is presented in Table 4

**Table 4:** Prognostic factors for complication (fibrosis ⩾Gr2) relapse-free survival (CRFS)

	**No tamoxifen *N*=57 (%)**	***P*-value**	**Tamoxifen *N*=90 (%)**	***P*-value**	**All patients *N*=147 (%)**	***P*-value**
CRFS rate (3 years)	66.4		47.8		54.0	0.0051
*Age, years*						
<60	60.7		52.2		55.3	
⩾60	79.4	0.20	43.2	0.25	53.1	0.46
*Histopathology*^a^						
IDC	64.8		46.3		52.6	
ILC	68.6		38.9		43.3	
DCIS	80.0		100.0		84.6	
Others	75.0	0.86	100.0	0.22	83.3	0.12
*TNM staging*						
T *in situ*	80.0		100.0		83.3	
T1	75.0		48.9		58.4	
T2	80.0		44.4		54.0	
T3/T4	33.3	0.26	22.2	0.30	28.2	0.08
N N0	75.6		52.1		62.9	
N1/N2/N3	58.3	0.27	38.5	0.28	43.6	0.04
M M0	70.6	—	45.9	—	55.5	—
*Type of surgery*						
Mastectomy	65.6		47.1		54.4	
Conservative						
Tumorectomy	50.8		53.1		51.4	
Quadrantectomy	85.7	0.31	37.5	0.41	55.3	0.93
*Margins*						
Clear	70.0		48.8		55.9	
Positive or close	30.0	0.15	25.0	0.39	29.6	0.23
*Menopausal status*						
Pre	87.5		45.5		63.2	
Peri or post	62.1	0.25	48.1	0.93	51.9	0.43
CD8⩽16	38.5		35.0		20.0	
16–24	70.3		64.5		66.2	
>24	76.1	0.002	81.8	<0.001	78.8	<0.001

a IDC=invasive ductal carcinoma; ILC=invasive lobular carcinoma; DCIS=ductal carcinoma *in situ*.

. CRFS rates were similar for all patient characteristics except for treatment with TAM and CD8 radiation-induced apoptosis. There was a statistically significant difference at 3 years in terms of CRFS rates: 48% (95% CI 37.2–57.6%) *vs* 66% (95% CI 49.9–78.6%) in the TAM and no-TAM groups, respectively. In each of these groups, CRFS rates were significantly lower for patients with low levels of CD8 radiation-induced apoptosis, 20% (95% CI 10–31.9%), 66% (95% CI 51.1–77.6%), and 79% (95% CI 55**–**90.9%) for CD8 ⩽16, 16–24%, and >24%, respectively.

### Complication-free survival

Complication-free survival according to prognostic factors is presented in Table 5

**Table 5:** Prognostic factors for complication (fibrosis ⩾Gr 2)-free survival (CFS) at 2 years

	**No tamoxifen *N*=57 (%)**	***P*-value**	**Tamoxifen *N*=90 (%)**	***P*-value**	**All patients *N*=147 (%)**	***P*-value**
CFS rate (2 years)	80.3		51.4		62.5	0.0007
Age, years						
<60	76.8		56.0		65.6	
⩾60	88.2	0.34	46.5	0.25	58.3	0.28
						
*Histopathology*^a^						
IDC	77.1		49.5		59.2	
ILC	100.0		44.4		59.7	
DCIS	80.0		100.0		84.6	
Others	75.0	0.61	100.0	0.27	83.3	0.26
*TNM staging*						
T *in situ*	80.0		100.0		83.3	
T1	77.8		48.9		59.2	
T2	90.0		50.4		61.5	
T3/T4	72.9	0.84	37.5	0.60	55.0	0.47
N N0	78.0		52.1		64.0	
N1/N2/N3	85.7	0.57	46.3	0.71	56.8	0.45
M M0	79.9	—	49.6	—	61.5	—
*Type of surgery*						
Mastectomy	90.9		61.4		74.0	
Conservative						
Tumorectomy	71.0		54.9		61.2	
Quadrantectomy	92.3	0.13	37.5	0.19	56.8	0.27
*Margins*						
Clear	80.4		52.0		62.6	
Positive or close	80.0	0.98	33.3	0.83	58.3	0.86
*Menopausal status*						
Pre	87.5		45.5		63.2	
Peri or post	79.1	0.59	52.2	0.88	62.5	0.83
CD8⩽16	46.1		14.7		23.1	
16–24	85.0		69.7		75.9	
>24	95.6	0.001	86.4	< 0.001	91.1	< 0.001

a IDC=invasive ductal carcinoma; ILC=invasive lobular carcinoma; DCIS=ductal carcinoma *in situ*.

. CFS rates were similar for all patient characteristics except for treatment with TAM and CD8 radiation-induced apoptosis. There was a statistically significant difference in CFS rates at 2 years, 51% (95% CI 40–61%) *vs* 80% (95% CI 67–89%) in the TAM and no-TAM groups respectively. In each of these groups, the CFS rates were significantly lower for patients with low levels of CD8 radiation-induced apoptosis: 23% (95% CI 12–36%), 76% (95% CI 61–85%), and 91% (95% CI 78–97%) for CD8 ⩽16, 16–24, and >24%, respectively. A multivariate analysis using the Cox proportional hazards regression model showed a significant increase in the risk of grade 2 or greater fibrosis in the group of patients treated with TAM, with a hazard ratio of 2.1 (95% CI 1.08–4.12, *P*=0.029), as well as in the group of patients considered as potentially more radiosensitive (CD8 apoptosis ⩽16) (Table 6

**Table 6:** Cox multivariate regression analysis for complication (fibrosis ⩾Gr 2)-free survival

**Prognostic variable**	**Hazard ratio**	**95% CI**	***P*-value**
CD8^a^ ⩽16	1		
16–24	0.22	0.11–0.43	<0.001
>24	0.08	0.03–0.24	<0.001
No tamoxifen	1		
Tamoxifen	2.1	1.08–4.12	0.029

a Percentage of radiation-induced CD8 lymphocyte apoptosis.

). The incidence of grade 2 or greater fibrosis was higher and at the limit of statistical significance in the group of patients with CD8 apoptosis ⩽16 treated with TAM, 31 out of 37 (84%) compared to no-TAM, seven out of 13 (54%). No grade 3 side effects were observed for patients with CD8 >24%.

Cumulative incidence rates for each failure type according to treatment are presented in Table 7

**Table 7:** A 2-year cumulative incidence of the first event (fibrosis ⩾Gr 2) and relapse according to CD8 radiation-induced apoptosis

	**No tamoxifen (%)**	**Tamoxifen (%)**	**All patients (%)**
Fibrosis ⩾Gr 2			
CD8⩽16	53.8	83.7	76
16–24	14.3	29.1	23
>24	4.3	13.6	8
Relapse			
CD8⩽16	7.7	2.7	4
16–24	9.5	6.5	7.7
>24	4.3	0	2.2

and Figure 1

**Figure 1:**
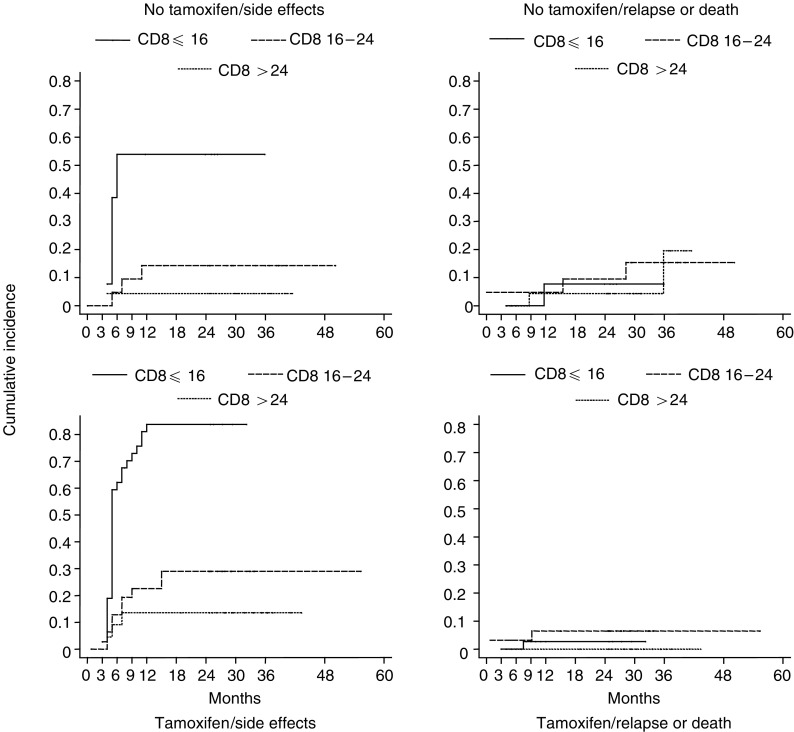
Cumulative incidence of grade 2 or greater fibrosis curves according to CD8 radiation-induced apoptosis and concomitant tamoxifen with radiation therapy.

. The 2-year complication-relapse-free survival rate was 54%, indicating that 46% of patients experienced either a grade 2 or 3 RTOG fibrosis, or relapse as a first event before 2 years (Table 4). For example, the 2-year cumulative incidence rates in the group of patients with CD8 T-lymphocyte apoptosis ⩽16 and treated with TAM was 83% for grade 2 or more fibrosis and 3% for relapse, which adds up to an overall incidence of 86%, the complement of the CRFS rate. In all patients, the relapse components were similarly distributed between the three categories of CD8, with an estimated cumulative incidence of 4, 7.7, and 2.2%, respectively.

## DISCUSSION

The concept that the inherent radiosensitivity of both normal cells and tumour cells varies from one individual to another is well established (Fertil and Malaise, 1981). This is clinically relevant because large patient-to-patient variation in radiation morbidity has been documented, even after RT with a fixed dose-fractionation schedule (Tucker *et al*, 1992; Bentzen *et al*, 1993; Bentzen, 1997). The data published so far on the cellular and molecular factors underlying acute or late tissue reactions appeared to be contradictory and suggest that there is no clear-cut relationship between cellular radiosensitivity and the risk of acute or late reactions; consequently, no test has been recommended up to now for predicting the risk or the severity of late reactions in breast cancer (Burnet *et al*, 1992; Brock *et al*, 1995; Jones *et al*, 1995; Johansen *et al*, 1996; Russell *et al*, 1998; Kiltie *et al*, 1999; Barber *et al*, 2000; Peacock *et al*, 2000; Oppitz *et al*, 2001). To confirm our first preclinical and retrospective studies on the correlation of radiation-induced CD4 and CD8 T-lymphocyte apoptosis (RTLA) and late side effects after RT (Ozsahin *et al*, 1997), we assessed prospectively RTLA by the prediction of individual intrinsic radiosensitivity of 399 consenting patients treated with curative RT for miscellaneous cancers (Ozsahin *et al*, 2003). RTLA significantly predicted grades 2 and 3 late effects (*P*<0.0001). Considering grade 3 late toxicity, patients with late effects (*n*=25) showed CD4 or CD8 radiation-induced apoptosis below the median (*P*<0.0001). The area under the curve of the receiver–operator characteristic curves of CD4 and CD8 apoptosis considered separately or CD4 and CD8 analyzed together were 0.84, 0.89, and 0.92, respectively (Ozsahin *et al*, 2003). To our knowledge, this is the first rapid predictive test based on lymphocyte apoptosis confirmed prospectively in a large number of patients. We considered CD8 more sensitive and more specific than CD4 T-lymphocyte apoptosis. We therefore analysed in the present study, the subgroup of 147 women who were treated for breast cancer by RT with (concomitant) or without TAM, and stratified by CD8 radiation-induced apoptosis.

Our finding of the influence of TAM on subcutaneous fibrosis, particularly in radiosensitive patients, is not supported by the results of any other published studies. Wazer *et al* (1992) showed a borderline significant trend, indicating an adverse impact of TAM on cosmetic appearance, but these preliminary results were not confirmed in their updated analysis (Wazer *et al*, 1997). Fowble *et al* (1996) reported on 154 patients treated with TAM and found no major adverse effect on cosmesis or complications. The timing of the TAM with the initiation of RT was unknown for 111 patients. In all, 23 patients received TAM during RT, and 20 began TAM after the completion of RT. Taylor *et al* (1995) showed that the use of adjuvant TAM did not appear to diminish the excellent cosmetic outcomes, irrespective of whether it was administered concurrently or sequentially with RT. In a recent prospective randomised study comparing breast pain after breast-conserving surgery and TAM with or without RT (Rayan *et al*, 2003), the incidence and severity of breast symptoms was similar at baseline in patients subsequently randomised to the RT and no-RT arms of the study. With lung fibrosis as an end point, contradictory reports have indicated that TAM could result in potentiation of postradiation normal tissue reactions (Bentzen *et al*, 1996; Huang *et al*, 2000; Koc *et al*, 2002; Mahler, 2002; Christensen *et al*, 2003).

The dominant cause of cosmetic failure in the patients treated with irradiation and TAM appeared to be retractive fibrosis. While not immediately apparent, there may be a mechanistic link between TAM therapy and postradiation normal tissue changes. TAM has been found to stimulate *in vitro* the secretion in human fibroblasts of the fibroblast mitogen, transforming growth factor *β* (TGF-*β*) (Colletta *et al*, 1990). There is evidence to suggest that the postradiation fibrotic response of normal tissues is, in part, mediated by TGF-*β*. Canney and Dean (1990) performed serial biopsies on irradiated and nonirradiated postsurgical patients. In four of the six irradiated patients, nontumour tissues within the treatment area stained positive for TGF-*β* beginning 9 weeks after therapy and continued to stain positive throughout the 40-week duration of the study. The nonirradiated patients showed no staining for TGF-*β* in non-tumour tissues. Therefore, if postradiation fibrosis is partially mediated by TGF-*β* and the secretion of this growth factor by fibroblasts is enhanced by TAM, then the presence of TAM may accentuate postradiation fibrosis. As postradiation changes in the breast may take years to stabilise (Beadle *et al*, 1984; Olivotto *et al*, 1989), such an interaction would not necessarily require the concomitant administration of RT and TAM. Finally, RT and TAM are both separately involved in initiating the skin fibrosis phenomenon by an increased production of the transforming growth factor-beta1 (TGF-*β*1) (Butta *et al*, 1992; Martin *et al*, 1993; Border and Noble, 1994), but it is not clear from our study whether the predominant effect of TAM is on the induction of RT injury or whether it is through a post-RT modification of the processing of RT injury in the tissue. We recommend delaying the start of TAM after completion of RT without reduced efficacy for the patients (Delozier *et al*, 1997), but interactions between both treatments may occur, even if they are separated in time (Parry, 1992; Bostrom *et al*, 1999).

In terms of local control, our data do not show any difference between the two groups of patients treated by RT alone (three patients) or concomitant RT+TAM (two patients). With a median follow-up of 29 months, the small number of local failures in our study lacks sufficient statistical power to allow the detection of a significant difference. Several retrospective studies have assessed the influence of TAM on local control and showed that TAM was associated with either no difference or a modest enhancement of local control (Rutqvist *et al*, 1992; Leborgne *et al*, 1995; Fowble *et al*, 1996; Christensen *et al*, 2003; Pierce *et al*, 2003). In these studies, the timing and sequencing of TAM administration relative to RT were either variable or not reported. The NSABP-B14 trial randomised 2644 patients with negative axillary lymph nodes between TAM and placebo. Breast-conserving surgery and RT were performed on 1072 patients, with TAM administered after surgery and during RT. There was a significant decrease in the breast relapse rate at 5 years with TAM (Fisher *et al*, 1989). More recently, Dalberg *et al* (1998) reported long-term results of adjuvant TAM in lymph node-negative postmenopausal women treated with breast-conserving surgery and postsurgical RT. The patients constituted a separate stratum of a larger trial, the Stockholm Adjuvant Tamoxifen Trial. TAM was started at the beginning of RT. In that study, the addition of TAM to RT resulted in a reduced rate of ipsilateral and contralateral breast tumour recurrences with a median follow-up of 8 years. The cosmetic toxicity over a long term was not mentioned by the authors.

Among the interesting questions arising from this study are whether subcutaneous fibrosis might be prevented, or at least reduced. First, our predictive radiation-induced lymphocyte apoptosis assay seems to be highly specific and sensitive to discriminate subgroups of patients as a function of their intrinsic radiosensitivity. Further prospective studies are still necessary before using this test in routine daily practice. Second, preliminary results have shown that TGF-*β* antagonists may inhibit or reduce the action of this growth factor (Border *et al*, 1990; Shah *et al*, 1992; Delanian *et al*, 1999; Lefaix *et al*, 1999; Delanian *et al*, 2003). The significant reduction of chronic RT damage obtained with the pentoxifylline and alpha-tocopherol combination (Delanian *et al*, 2003) does not support the concept that established RT sequelae such as radiation-induced subcutaneous fibrosis are irreversible. Third, evidence from the first analysis of the ATAC (Arimidex, Tamoxifen Alone or in Combination Trialists Group) trial supports the use of aromatase inhibitors such as anastrozole for the adjuvant treatment of early breast cancer in postmenopausal women (Baum *et al*, 2002). Our data show that in radiosensitive patients, TAM should be delayed after completion of RT. Another approach could be to replace TAM by an aromatase inhibitor. This type of molecule has yet to be tested concomitantly with RT in a clinical setting. Recently, we demonstrated the radiosensitisation of breast cancer cells transfected with the aromatase gene by the nonsteroidal aromatase inhibitor letrozole (Azria *et al*, 2003).

We conclude that the concomitant use of TAM with RT is significantly associated with the incidence of subcutaneous fibrosis but not telangiectasia. In patients receiving adjuvant hormonal treatment, TAM and RT should only be administered concomitantly with caution to radiosensitive patients.
